# Altered Resting-State Functional Connectivity of the Striatum in Parkinson's Disease after Levodopa Administration

**DOI:** 10.1371/journal.pone.0161935

**Published:** 2016-09-09

**Authors:** Wanqun Yang, Bin Liu, Biao Huang, Ruiwang Huang, Lijuan Wang, Yuhu Zhang, Xiong Zhang, Kai Wu

**Affiliations:** 1 Department of Radiology, Guangdong Academy of Medical Sciences, Guangdong General Hospital, Guangzhou, Guangdong, P.R. China; 2 Center for the Study of Applied Psychology, Key Laboratory of Mental Health and Cognitive Science of Guangdong Province, School of Psychology, South China Normal University, Guangzhou, Guangdong, P.R. China; 3 Department of Neurology, Guangdong Academy of Medical Sciences, Guangdong General Hospital, Guangzhou, Guangdong, P.R. China; 4 Department of Biomedical Engineering, School of Materials Science and Engineering, South China University of Technology, Guangzhou, P.R. China; 5 Department of Nuclear Medicine and Radiology, Institute of Development, Aging and Cancer, Tohoku University, Sendai, Japan; Institute of Psychology, Chinese Academy of Sciences, CHINA

## Abstract

**Background:**

Despite improvement in motor symptoms, the effect of dopaminergic medications on cognition in patients with Parkinson’s disease (PD) is less clear. The purpose of this study was to reveal levodopa-induced acute changes in the functional connectivity of the striatum in patients with PD compared with matched untreated patients and healthy volunteers.

**Methods:**

Twenty-two patients with PD underwent functional magnetic resonance imaging both ON and OFF dopamine-replacement therapy on two consecutive days. Twenty-eight normal aging volunteers also did them without taking in levodopa. Three caudate seeds and two putamen seeds were selected to calculate functional connectivity intensity.

**Results:**

Motor symptoms measured by UPDRS were significantly worse in PD OFF than PD ON. Decreased functional connectivity in PD OFF compared to controls was detected in the following seed regions: dorsal caudate, ventral putamen and dorsal putamen. Increases in connectivity in PD ON compared to controls were found in the primary and supplementary motor areas and the associative prefrontal and parietal regions, while decreases in anterior cingulate, ventromedial prefrontal cortex, and parahippocampal gyrus. For the ventral striatal seeds, decreased connectivity in PD ON compared to PD OFF was found in the ventromedial prefrontal and orbitofrontal regions, dorsolateral prefrontal regions. For the dorsal striatal seeds, increased connectivity in PD ON compared to PD OFF was observed in the primary and secondary motor areas.

**Conclusion:**

Our results suggest that levodopa significantly changes the motor and cognitive networks of the cortico-striatal pathways. This knowledge will lead clinicians to survey a broader range of symptoms in determining optimal therapy.

## Introduction

Parkinson’s disease (PD) is an aging-related progressive neurodegenerative disorder characterized by resting tremor, rigidity, bradykinesia and postural instability, and gait disturbances. The reported incidence of PD is 112.5 per 1,000 patient-years, and PD is one of the main causes of disability in the elderly [1]. Pathologically, degeneration of dopaminergic cells in the pars compacta of the substantia nigra results in dopamine depletion in the striatum, and then dysfunction of cortico–striatal–thalamic loops ensues [2]. The ventral tegmental area which dopaminergic innervation is on the ventral striatum, however, is relatively spared in PD patients. Dopamine replacement has become the primary therapy in PD patients to compensate for the loss of dopamine and excessive dopamine might occur in ventral striatum. The overdose of dopamine in ventral striatum may alter activity of the related brain regions, and may be associated with cognitive dysfunction [3–5]. The prevalence of dementia in patients with PD is close to 30%, and the incidence is 4 to 6 times greater than that of the general age-appropriate population [1]. Therefore, further research of the neurophysiological variations between before and after levodopa administration in patients with PD is needed.

The effect of dopaminergic medications on cognition in PD is less clear, despite it could improve motor symptoms of PD patients. Functional connectivity analysis of resting-state functional magnetic resonance imaging (fMRI) has recently provided new insights into networks in human brain. Di Martino et al. [6] has demonstrated different functions mediated by the ventral and dorsal striatum in healthy adults. In PD patients, dopamine depletion leads to a remapping of cerebral connectivity that reduces the spatial segregation between different cortico-striatal loops[7]. In another study, Yang et al. [8] reported cognitive decline may be associated with abnormal spontaneous brain activity occurring in the occipital regions and associative cortical areas in early PD patients. Another fMRI study [9] has demonstrated, dopamine replacement improves dorsal striatum-mediated motor symptoms but impairs ventral striatum function in PD patients, but this study used task related fMRI without examining acute changes between ON and OFF medication states. Krajcovicova et al. [10] reported no significant difference in the resting-state network integrity between cognitively normal PD patients on dopaminergic medications and healthy people. But another study comparing ON and OFF medication states in PD patients and age-matched controls showed that cognitive and motor circuitries were less separable in older adults than in healthy younger adults [11].

The aim of this study was to evaluate levodopa-induced acute changes in the resting state functional connectivity of the striatum in PD patients. Based on previous studies, we hypothesis that PD patients on the ON medication state would show increased functional connectivity on motor circuitries compared to PD patients on the OFF state; however, due to overdose of dopamine in ventral striatum, cognitive networks would be altered in PD patients after levodopa administration.

## Materials and Methods

### Participants

Between September 2012 and July 2014, twenty-seven patients with PD and thirty-nine age- and gender-matched healthy controls were recruited in this study. PD was diagnosed by an experienced neurologist according to the UK Parkinson's Disease Society Brain Bank Clinical Diagnostic Criteria. All patients were evaluated with the Unified Parkinson's Disease Rating Scale (UPDRS) [12], Mini-Mental State Exam (MMSE) [13], Montreal Cognitive Assessment (MOCA) [14], and the Hoehn and Yahr scale[15] in an OFF medication state. The inclusion criteria for PD patients were (1) MMSE score > 23; (2) Hoehn and Yahr stage in 1–2.5; (3) Hachinski Ischemic Score (HIS) [16]< 4; (4) no history of any psychiatric or neurological disease; and (5) MRI revealed only cerebral atrophy and little T2-weighted high signal in the deep white matter (maximum diameter < 1 cm). The exclusion criteria were (1) acute cerebrovascular disease history in the recent 3 months; (2) active epilepsy; (3) history of mental illness such as delirium, depression, or anxiety disorders; (4) diagnosed with atypical Parkinsonian disorders or secondary Parkinson syndrome; and (5) severe claustrophobia or contraindications to MRI (e.g., pacemaker, metallic foreign bodies). All healthy controls were assessed with the MMSE and Hoehn and Yahr scale. The inclusion criteria for the controls were (1) normal neurological examination; (2) no history of any psychiatric or neurological disease; (3) normal MRI examination except for cerebral atrophy and little T2-weighted high signal in the deep white matter (maximum diameter < 1 cm); (4) MMSE score ≥ 28; and (5) Clinical Dementia Rating (CDR) [17] was 0. Four PD patients and four healthy controls were excluded because of large head motions (> 1.5 mm) in the z direction. One PD patient and six healthy controls were excluded due to severe MRI artifacts and frontal lobe deformation caused by the frontal sinus. One healthy control was excluded because the subject could not tolerate the whole fMRI scan.

The remaining 22 PD patients (61.58 ± 9.06 years; 9 M / 13 F) and 28 healthy controls (60.64 ± 4.58 years; 13 M / 15 F) were included in the analysis. All subjects were right-handed. The demographic and clinical characteristics of the patients and controls are shown in Table 1. Akinesia was the predominant symptom, which was dominant on the binary sides in 13 patients, on the right side in 5 patients and on the left side in 4 patients. All patients had an obvious delay in movement initiation and a mild tremor. The study protocol was approved by Guangdong General Hospital, Guangdong Academy of Medical Sciences research ethics committee. Written informed consent was obtained from each subject prior to participation this study.

**Table 1:** Demographic and clinical characteristics of participants (mean ± SD).

Parameter	Parkinson’s disease (n = 22)	Control (n = 28)	*P* Value
Age	61.58±9.06	60.64±4.58	>0.05
Gender (M/F)	9/13	13/15	>0.05
Education (Y)	11.26±3.142	11.45±3.648	>0.05
Disease duration (Y)	3.76±2.79	**—**	
H&Y stage OFF	1.84±0.67	**—**	
Symptom-dominant side (bilateral/right/left)	13/5/4	**—**	
UPDRS III OFF*	32.16±9.36	**—**	
UPDRS III ON*	24.53±7.40	**—**	
MMSE	24.26±2.75	29.05±1.430	<0.05
SDS	43.25±7.09	41.37±6.75	>0.05
SAS	44.50±8.27	42.69±7.23	>0.05
SCL-90 Score	142.50±39.65	139.96±38.76	>0.05
HIS	2.00±1.054	0.23±0.528	>0.05
L-dopa (mg)	400	**—**	

Abbreviations: UPDRS III, Unified Parkinson’s Disease Rating Scale motor score; H&Y, Hoehn and Yahr staging; MMSE, Mini-Mental State Examination; SDS, Self-Rating Depression scale; SAS, Self-Rating Anxiety Scale; SCL-90 Score, Symptom Check List-90; HIS, Hachinski Inchemic Score; F, female; M, male; Y, year. L-dopa = daily L-dopa equivalent dose in milligram

OFF = OFF medication; ON = ON medication.

*: The UPDRS motor score OFF medication was significantly higher than that ON medication (*t* = 2.789, *P* = 0.008).

### Procedure

Each PD patient underwent two fMRI sessions over two consecutive days corresponding to the ON and OFF medication states respectively. The ON medication state was defined as 60–90 minutes after the patient received a dose of 400 mg levodopa, and the OFF state was withdrawal from medication for more than 12 hours (The patients took the levodopa before 7:00 pm the day before the testing day to avoid disturbing for clinical therapy). Each healthy control underwent the same procedure without any medication. In order to eliminate the bias due to patient fatigue and baseline drift of the MRI signal, the fMRI scans for the healthy controls were completed at the same time points corresponding to the ON and OFF medication states over two consecutive days as with the PD patients.

### MRI Acquisition

The resting-state fMRI data were acquired using a 3.0 T MRI scanner (Signa Excite II HD GE Healthcare, Milwaukee, WI). During the resting-state fMRI scan, every subject was instructed to keep their eyes closed, not to think of anything in particular, and not to fall asleep. During scanning, the subject was positioned supine in the gantry of the scanner with foam padding to limit head movement, and ear plugs to reduce the impact of acoustic noise. The resting-state fMRI data were obtained by using a gradient-echo echo planer imaging (GRE-EPI) sequence with the following parameters: repetition time/echo time (TR/TE) = 2000/30 ms, field of view (FOV) = 240 mm × 240 mm, data matrix = 64 × 64, flip angle (FLA) = 80°, in-place resolution = 3.75 mm × 3.75 mm, slice thickness = 4 mm, inter-slice space = 1mm, and 30 axial slices covering the whole brain. For high resolution of brain structural images, we adopted a T1-weighted fast spoiled gradient recalled echo inversion recovery (FSPGRIR) sequence (TR = 7.6 ms, TE = 3.4ms, FOV = 240 mm × 240 mm, matrix = 256 × 256, FLA = 13°, voxel size = 0.94 mm × 0.94 mm, slice thickness = 1 mm, inter-slice space = 0, and 146 sagittal slices).

### Data preprocessing

The resting-state fMRI data were analyzed using DPARSF (Data Processing Assistant for Resting-state fMRI, http://www.restfmri.net), which is based on SPM8 (Statistical Parametric Mapping, Version 8, Welcome Department of Cognitive Neurology) implemented in Matlab. We performed preprocessing in following steps: (1) discarding the first 10 time points of fMRI data for each subject to eliminate the impact of magnetization equilibrium, (2) slice-time correction, (3) head motion correction by aligning to the first image of each session (head movements were limited to less than 1.5 mm in translation and 1.5° in rotation), (4) spatial normalization to a Montreal Neurological Institute (MNI) standard space (EPI MNI-152 space), (5) smoothing with a Gaussian filter of 6 mm full width at half maximum (FWHM), (6) time series detrending and normalization to a zero mean and a unit variance, (7) temporary band-pass filtering (0.01 ~ 0.08 HZ) to remove low-frequency drifts and physiological high-frequency noise, (8) data masking to exclude non-brain voxels, and (9) extracting the time course of activity from the striatal seed regions.

**Fig 1:**
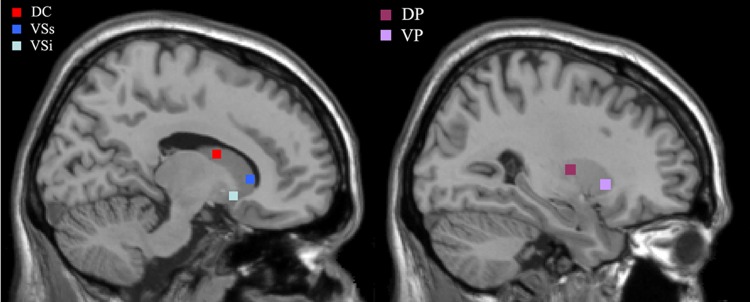
Representation of the five striatal seed regions. The left sagittal brain shows the locations of the three caudate seeds: inferior ventral striatum (VSi), superior ventral striatum (VSs), and dorsal caudate (DC). The right shows the locations of the two putamen seeds: dorsal putamen (DP) and ventral putamen (VP).

Because the distinct function of ventral and dorsal striatum and different degrees of dopamine depletion in ventral and dorsal striatum, we selected five regions in the striatal area as seed regions, with three regions in ventral striatum and two in dorsal striatum. The regions and their MNI x, y, z coordinates were as follows: inferior ventral caudate (VCi (±) 9 9−8), superior ventral caudate (VCs (±) 10 15 0), dorsal caudate (DC (±) 13 15 9), ventral putamen (VP (±) 20 12−3) and dorsal putamen (DP (±) 25 8 6) (Fig 1). According to the shape and size of the caudate and putamen, a four voxels square on the sagittal image was placed around these coordinates for each seed region, all the seed regions were selected in accordance with previous studies[6,11]

### Functional connectivity and statistical analyses

The extracted time course of each seed region was used to correlate with all other voxels in the whole brain to form functional connectivity maps in each subject, which was carried out by REST (Resting-state fMRI Data Analysis Toolkit, http://www.restfmri.net). Fisher *r*-to-*z* transform was used to normalize the correlation coefficient [z = 0.5 log (1+*r*)/(1-*r*), *r* is the correlation coefficient]. We applied group-level random effects analysis to Z scores from all subjects in each group by using REST. First, One sample *t* test was used to determine statistical differences in functional connectivity with each seed of striatum in controls, PD OFF and PD ON group, with a threshold of *p* < 0.001 (AlphaSim correction and an extent voxel threshold of 6). The correction thresholds were determined by Monte Carlo simulations with the program AlphaSim in AFNI. Then, comparisons of functional connectivity between ventral striatum versus dorsal striatum in either PD OFF or PD ON to the control group were done using student's two-sample *t*-test. At last, in order to invest how the levodopa influenced functional connectivity by comparing the differences of functional connectivity between the PD OFF and PD ON patients, we compared the difference between the PD OFF and PD ON states using the paired *t*-test. In the calculations, we regressed out the effects of age, gender, education, and head motion. A threshold of *p* < 0.05 with AlphaSim correction and an extent voxel threshold of 85 were selected for determining the significant between-group difference in connectivity maps.

To further assess the specific functional network of the ventral and dorsal striatum, we also combined the three ventral striatum seed regions (inferior ventral caudate, superior ventral caudate, and ventral putamen) together and combined the two dorsal striatum seed regions (dorsal putamen and dorsal caudate) together (similar approach can be seen in the literature [11]), using REST Image Calculator. Comparison of the ventral and dorsal striatal connectivity maps were calculated in PD OFF, PD ON, and controls separately using a threshold of *p* < 0.001 (AlphaSim correction and an extent voxel threshold of 6).

## Results

### Clinical and motor evaluation

The modified Hoehn and Yahr score ranged between 1 and 2.5 (1.84 ± 0.67). Motor symptoms evaluated by UPDRS were remarkably worse in the PD OFF than PD ON medication state (*t* = 2.789, *p* <0.01). This result demonstrated that levodopa obviously improved motor functioning of PD patients, and the improvement was most remarkable in the more affected side. The average disease duration of PD, as an indirect marker of the disease severity, was 3.76 ± 2.79 years (Table 1). No significant difference in gender, age and educational level were noted between PD patients and controls (*p >* 0.05).

### Functional connectivity with each seed of striatum in controls, PD OFF and PD ON group

We first displayed each seed of striatum functional connectivity maps for the control group, PD OFF, and PD ON groups to evaluate the anatomical appearance of the networks (*p* <0.001, AlphaSim, *K* ≥ 6 voxels). The results were in accordance with previous findings of distinct cognitive, motor, and reward cortico-striatal connectivity within striatal subregions [6,18]. In all three groups, inferior ventral striatum and superior striatum showed connectivity with ventral medial prefrontal areas and anterior cingulate cortex, while dorsal caudate showed connectivity with areas more dorsal and lateral in prefrontal areas. The two ROIs of putamen mainly showed connectivity with the primary and supplementary motor area. However, the specificity of the functional connectivity patterns of the two putamen seed regions was decreased in both PD OFF and PD ON groups, because the two ROIs of putamen also showed connectivity with prefrontal areas, cingulate cortex and supramarginal area except for sensorimotor areas (S1, S2 and S3 Tables and S1, S2 and S3 Figs).

### Comparison of functional connectivity for each seed of striatum between controls, PD OFF and PD ON group

**Fig 2:**
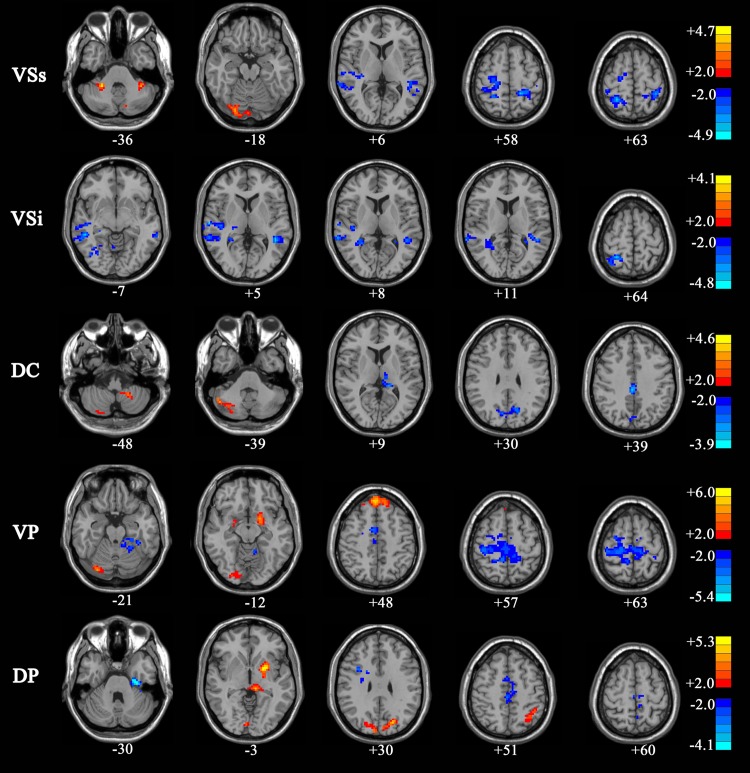
Regions showing significant functional connectivity with the striatal seeds in PD OFF compared with normal control subjects (two-sample *t* test, *P* <0.05, AlphaSim, K ≥85 voxels).

Decreased functional connectivity in PD OFF versus controls was observed in the following regions: anterior cingulate gyrus, right superior temporal gyrus, middle temporal gyrus, precentral gyrus, postcentral gyrus, superior parietal lobule, paracentral lobule, and supplementary motor area (AlphaSim, *p* < 0.05, K ≥ 85 voxels). Connectivity between the dorsal caudate and the right cuneus gyrus, and connectivity between the dorsal putamen and the left fusiform gyrus were decreased in PD OFF compared to controls. Increased functional connectivity in PD OFF compared to controls was noted in these regions: left putamen, right lingual gyrus, right medial frontal gyrus, and bilateral cerebellum. The results also indicate that the compensatory increased connectivity with the cerebellum and pons occurs in PD patients when cortex-striatum loop connectivity is decreased. For the inferior ventral caudate, there were no regions that showed greater connectivity in PD OFF than controls (S4 Table and Fig 2).

Increases in connectivity in PD ON compared to controls were found in the primary and supplementary motor areas and the associative prefrontal and parietal regions, while decreases in anterior cingulate, ventromedial prefrontal cortex, and parahippocampal gyrus (S5 Table and S4 Fig).

**Fig 3:**
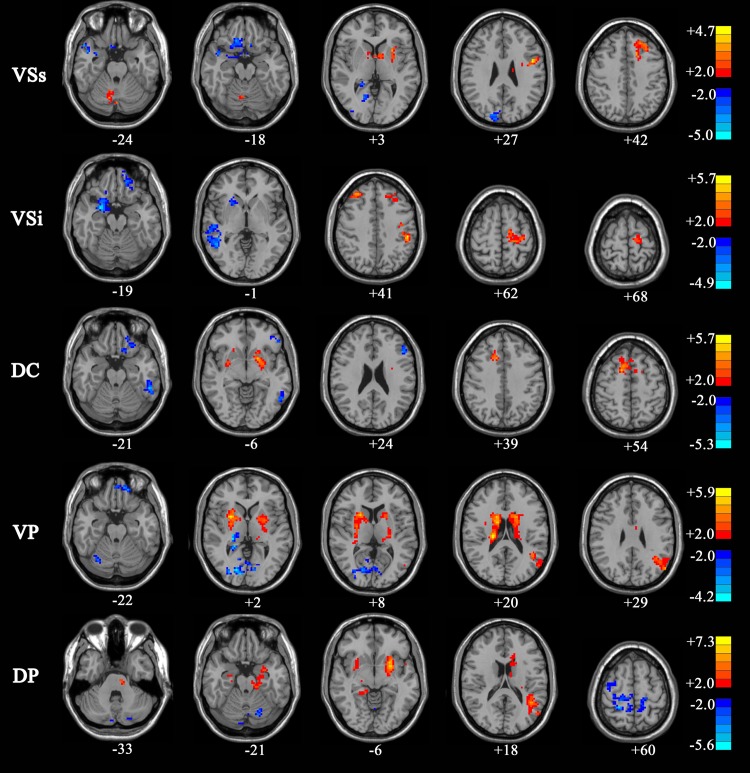
Regions showing significant functional connectivity with the striatal seeds in PD ON compared with PD OFF (two-sample *t* test, *P* <0.05, AlphaSim, K ≥85 voxels). The regions were overlaid on anatomical images from a representative subject of the MNI series. “Hot” colors indicate increased correlation strength in PD ON and “Cold” colors indicate decreased correlation strength in PD ON relative to PD OFF. VCi, inferior ventral striatum; VCs, superior ventral striatum; DC, dorsal caudate; VP, ventral putamen; DP, dorsal putamen.

In general, increased cortico-striatal functional connectivity was mainly located in the frontal lobe, temporal lobe, precentral gyrus, paracentral lobule, SMA, angular gyrus and cerebellum in PD ON patients compared to PD OFF, while decreased functional connectivity was mainly located in the temporal lobule, limbic lobule, hippocampus gyrus, parahippocampal gyrus and cingulate gyrus and amygdala (AlphaSim, *p* < 0.05, K ≥85 voxels) (Table 2 and Fig 3).

**Table 2:** Significant connectivity difference between PD OFF and PD ON.

Region	Voxel	MNI coordinates			*T*-value
		X	Y	Z	
**PD on>PD off**					
**Superior ventral striatum (VSs)**					
Cerebelum_Crus2_L	30(87)	-9	-78	-33	3.8537
Precentral_L	54(139)	-48	0	27	4.4659
Frontal_Mid_L	93	-27	36	45	4.7338
Frontal_Sup_L	146				
**Inferior ventral striatum (VSi)**					
Frontal_Mid_R	79(101)	27	36	39	4.5546
Frontal_Mid_L	85	-27	36	51	4.0236
Parietal_Inf_L	53(119)	-51	-30	39	5.7434
Precentral_L	111	-15	-15	75	4.0673
Paracentral_Lobule_L	58*				
**Dorsal caudate (DC)**					
Supp_Motor_Area_R	88				
Frontal_Sup_R	72*	15	9	54	4.529
**Ventral putamen (VP)**					
Pallidum_L	35*				
Angular_L	120	-54	-72	27	4.1401
**Dorsal putamen (DP)**					
Cerebelum_6_L	51(173)	-18	-87	-45	-4.3636
Precentral_R	47(148)	39	6	42	-3.6596
Postcentral_R	96	24	-42	63	-5.6279
Precuneus_L	36(90)	-18	-27	57	-3.758
Paracentral_Lobule_L	48(566)				
**PD on <PD off**					
**Superior ventral striatum (VSs)**					
Temporal_Pole_Mid_R	44(117)	45	15	-30	-3.766
Rectus_R	40(247)	9	12	-27	-4.0716
Lingual_R	67(111)	18	-51	-3	-4.9856
Cuneus_R	26(93)	12	-87	24	-3.7651
Limbic Lobe	41(247)				
Occipital_Mid_R	30(93)				
**Inferior ventral striatum (VSi)**					
Amygdala_R	26(285)	27	0	-18	-4.8943
Parahippocampa Gyrus R	31(285)				
Frontal_Sup_Orb_L	43(95)	-18	39	-21	-3.7469
Temporal_Mid_R	109	48	-54	-3	-4.2505
**Dorsal caudate (DC)**					
Frontal_Sup_Orb_L	24(121)	-18	39	-24	-3.3495
Temporal_Inf_L	112	-54	-45	-18	-5.3074
Temporal_Mid_L	36(151)				
Frontal_Inf_Tri_L	80(104)	-51	24	27	-5.0956
Frontal_Sup_Orb_L	24(121)	-18	39	-24	-3.3495
**Ventral putamen (VP)**					
Frontal_Sup_Orb_L	26(92)	-12	48	-24	-4.1462
Calcarine_R	86	18	-81	3	-4.1923
Lingual_R	118				
Thalamus_R	10*(44)	24	-30	3	-4.1275
Precuneus_R	51*	6	-45	54	-3.2013
Frontal_Sup_Orb_L	26(92)	-12	48	-24	-4.1462
**Dorsal putamen (DP)**					
Hippocampus_L	41*				
Pallidum_L	36*				
ParaHippocampal_R	30*				
Temporal_Mid_L	73*	-42	-51	18	5.3552
Cingulate GyrusL	40(129)	-9	-3	21	4.4251

Note: The comparison of the brain regions showing significant difference between PD on medication and PD off medication (P <0.05, AlphaSim, K ≥85 voxles).

* indicates that the cluster don’t reach the preset threshold value. T indicates the correlation intensity. The coordinates are given as stereotaxic coordinates referring to the atlas of MNI. L, left; R, right.

### Comparison of functional connectivity between ventral striatum combined versus dorsal striatum combined within controls, PD OFF and PD ON group

**Fig 4:**
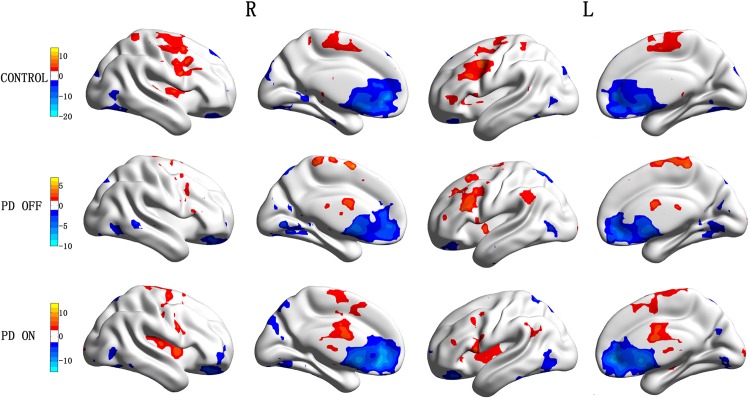
Regions showing greater connectivity with ventral striatal seeds (VCi, VCs, and VP combined) than with dorsal striatal seeds (DC and DP combined) in “Cold” and regions showing greater connectivity with dorsal striatal seeds than with ventral striatal seeds in “Hot” (*P* <0.001, AlphaSim, *K* ≥6 voxels).

In the control group, the regions showing greater correlation with the ventral striatum seeds than the dorsal striatum seeds included the bilateral inferior occipital gyrus, parahippocampal gyrus, and superior frontal gyrus, while the dorsal striatum seeds than the ventral striatum seeds included the cerebellum posterior lobe, midbrain, bilateral caudate, putamen, thalamus and precentral gyrus, inferior frontal gyrus, middle frontal gyrus, superior frontal gyrus, SMA, and medial frontal gyrus (pre-SMA) (*p* < 0.001, AlphaSim, *K* ≥ 6 voxels) (Table 3 and Fig 4). The results are consisted with previous findings that the ventral striatum is mainly involved in the cognitive network, while the dorsal striatum is mainly involved in the motor network.

**Table 3:** Direct comparisons between ventral striatum seeds combined and dorsal striatum seeds combined.

Contrast	Region	Voxel		MNI coordinates			*T*-value
				X	Y	Z	
Control						
**Ventral >dorsal**	L Parahippocampal	4*	-15	0	-24	-4.8871
	L Inferior Occipital Gyrus	7	-30	-81	-12	-5.9254
	R Inferior Occipital Gyrus	8	36	-78	-12	-4.6388
	R Superior Frontal Gyrus	29	15	57	39	-5.8457
**Ventral <dorsal**	R Cerebellum Posterior Lobe	6	48	-66	-42	4.1311
	L Cerebellum Posterior Lobe	20	-30	-90	-36	5.2896
	Midbrain	5*	-3	-33	-3	5.0991
	R Inferior Frontal Gyrus	30	51	18	27	5.4477
	L Inferior Frontal Gyrus	11	-45	12	9	5.2371
	L Precentral Gyrus	52	-42	6	45	7.9044
	R Middle Frontal Gyrus	67	45	9	51	6.3679
	L Middle Frontal Gyrus	28	-33	6	57	7.1318
	R SMA	18	6	-6	54	4.6229
	L SMA	12	-6	6	51	5.2001
	R Superior Frontal Gyrus	6	30	0	63	4.1732
**PD OFF**						
**Ventral >dorsal**	L Lingual Gyrus	19	-24	-63	-9	-5.1078
	R Lingual Gyrus	23	15	-78	-3	-5.8494
	L Middle Occipital Gyrus	31	-21	-84	12	-5.9675
	L Superior Parietal Lobule	32	-24	-60	66	-6.1041
**Ventral <dorsal**	L Cerebellum Posterior Lobe	8	-24	-93	-36	4.6977
	R Middle Frontal Gyrus	8	45	12	45	4.5054
	L Middle Frontal Gyrus	11	-24	27	39	4.6239
	R SMA	13	9	-3	66	4.6715
	L SMA	52	-3	0	66	8.6884
	L Medial Frontal Gyrus (pre-SMA)	8	0	30	48	6.2636
	R Paracentral Lobule	23	3	-33	66	5.7572
**PD ON**						
**Ventral >dorsal**	R Superior Parietal Lobule	15	24	-66	60	-7.6956
	L Superior Parietal Lobule	9	-21	-57	63	-5.8605
**Ventral <dorsal**	Pons	6	0	-33	-33	4.552
	Cerebellum Posterior Lobe	6	-33	-90	-27	6.1556
	R Precentral Gyrus	16	57	3	33	4.9209
	R SMA	6	6	-15	78	4.4268
	L SMA	25	0	3	57	5.1347
	L Middle Frontal Gyrus	12	-36	6	63	7.95
	R Superior Frontal Gyrus	22	27	-6	66	5.5019

Note: The comparison of the brain regions showing significant difference between ventral striatum seeds combined and dorsal striatum seeds combined in each group (*P* <0.001, AlphaSim, *K* ≥6 voxels).

* indicates that the cluster don’t reach the preset threshold value. The coordinates are given as stereotaxic coordinates referring to the atlas of MNI. L, left; R, right.

In PD OFF patients, the regions show greater correlation with the ventral striatum seed than the dorsal striatum seed included the bilateral lingual gyrus and left superior parietal lobe, while the dorsal striatum seeds than the ventral striatum seeds included the bilateral putamen, middle frontal gyrus, SMA, and right paracentral lobule (*p* < 0.001, AlphaSim, *K* ≥ 6 voxels) (Table 3 and Fig 4). In addition, the inferior ventral caudate showed connectivity with the rectus gyrus and medial frontal gyrus, and the ventral putamen showed connectivity with the superior temporal gyrus.

In PD ON patients, the dorsal striatum showed greater connectivity with the medial frontal gyrus, insula and right precentral gyrus, and left middle frontal gyrus. The dorsal caudate showed connectivity with the right angular gyrus, while the dorsal putamen showed connectivity with the right supra marginal gyrus (*p* < 0.001, AlphaSim, *K* ≥ 6 voxels). The ventral striatum showed greater connectivity with the anterior cingulate, and left superior medial frontal gyrus (Table 3 and Fig 4). These results indicate that in both PD OFF and PD ON patients, functional connectivity patterns of the ventral striatum and dorsal striatum are different.

### Comparison of functional connectivity maps of ventral striatum combined and dorsal striatum combined between PD OFF and PD ON patients

Connectivity maps for PD ON versus PD OFF were compared (AlphaSim, *p* < 0.05, K ≥ 85 voxels). For the dorsal striatum seeds, increased connectivity in PD ON compared to PD OFF was found in the bilateral putamen. The connectivity between the dorsal caudate and the right supplementary motor area and superior frontal gyrus, and connectivity between the dorsal putamen and the left hippocampus and right parahippocampal gyrus was increased in PD ON compared to PD OFF. Decreased connectivity in PD ON compared to PD OFF was found in the bilateral temporal lobe.

**Fig 5:**
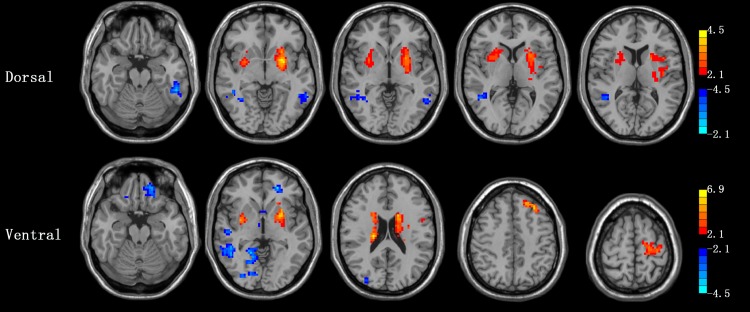
Regions showing greater connectivity with ventral striatal seeds (VCi, VCs, and VP combined) and with dorsal striatal seeds (DC and DP combined) in PD ON than PD OFF in “Hot”; Regions showing greater connectivity with ventral striatal seeds and with dorsal striatal seeds in PD OFF than PD ON in “Cold” (P <0.05, AlphaSim, K ≥85 voxels).

For the ventral striatum seeds, increased connectivity in PD ON compared to PD OFF was observed in the left precentral gyrus and left frontalis medius gyrus. The functional connectivity between the cerebellum and superior ventral caudate, between the inferior parietal lobule and inferior ventral caudate, and between the left angular gyrus and ventral putamen were observed. Decreased connectivity in PD ON compared to PD OFF was found in the left superior frontal gyrus, right rectus, right lingual gyrus, inferior frontal gyrus, medial frontal gyrus, right occipital lobe, temporal lobe, limbic lobe, and calcarine area (Table 4 and Fig 5). These results indicate that levodopa normalized functional connectivity in PD, with specific effects on the functional networks associated with dorsal striatum seeds.

**Table 4:** Significant connectivity difference between PD on medication and PD off medication.

Region	Voxel	MNI coordinates			*T*-value
		X	Y	Z	
**Dorsal striatum**					
**PD on > PD off**					
Putamen_L	174	-24	12	-9	7.8762
Putamen_R	105	30	0	-6	4.2201
**PD on < PD off**					
Temporal_Inf_L	100	-54	-45	-18	-5.413
Temporal_Mid_R	26*	51	-51	6	-4.3812
Precentral_L	116	-24	-21	60	3.7206
Frontal_Mid_L	90	-30	30	45	4.5343
**Ventral striatum**					
**PD on > PD off**					
Precentral_L	116	-24	-21	60	3.7206
Frontal_Mid_L	90	-30	30	45	4.5343
**PD on < PD off**					
Frontal_Sup_Orb_L	54*	-21	45	-6	-4.6445
Rectus_R	32*	12	21	-12	-4.2994
Lingual_R	107	18	-51	-6	-4.6583
Occipital_Mid_R	49*	33	-81	9	-3.3916
Temporal_Mid_R	91	48	-45	9	-4.7732

Note: The comparison of the brain regions showing significant difference between ventral striatum seeds combined and dorsal striatum seeds combined in PD on medication versus PD off medication (P <0.05, AlphaSim, K ≥85 voxles).

* indicates that the cluster don’t reach the preset threshold value. T indicates the correlation intensity. The coordinates are given as stereotaxic coordinates referring to the atlas of MNI. L, left; R, right.

## Discussion

Areas with decreased connectivity we found in PD OFF patients compared to controls, are the so-called limbic system and its associative regions. However, there have been few reports of the relationship with limbic system. Notably, the cognitive function in our PD patients was impaired, that is, dopamine depletion in our PD patients involved in not only the nigral-striatum dopamine pathway but also the limbic midbrain system, and the nigral-striatum took a more part effect of Parkinson’s disease. It appears that cognitive and motor circuitries are less separable, which is consistent with the finding of Kwak et al. [11].

Our study showed that the connectivity with cerebellum and pons compensation increased after cortex-striatum loop connectivity decreased in patients with PD. However, this compensation will be increased after dopaminergic medication. That is, dopamine replacement can change the compensating mechanism, consequently affecting cognitive activities. Although levodopa is not an effective treatment for PD cognitive dysfunction, the change of connectivity of the ventral and cerebellum and pons can serve as an observable biomarker of the cognitive response to treatment. Ultimately, this knowledge may lead clinicians to consider a broader range of symptoms in adjusting medication dosages to strike a better balance. Recent studies also have revealed that the putamen may receive projections from the cerebellum via the thalamus. Hacker et al. [2] have suggested that striatal functional connectivity with the brainstem is graded (posterior putamen > anterior putamen > caudate), corresponding to well-documented gradient of striatal dopaminergic function loss in PD. These disrupted putamen-cortical and putamen-cerebellar loops and brainstem may explain why PD patients gradually develop motor disorders such as tremor, rigidity, and difficulties with balance, sleep disturbance, and dysautonomia, all of which suggest dysfunction of the pedunculopontine nucleus. Additionally, a study by Wu et al. [19] indicated that connectivity between the putamen and these areas was negatively correlated with UPDRS motor scores, suggesting that as the disorder progresses these interregional interactions become weaker. However, according to Braak [20] the cerebral distribution of Lewy body histopathology, even at late disease stages, does not correspond to the presently observed putamen-cortical functional connectivity alterations, and the sensorimotor and visual cortices are the last affected regions. Yet Wu et al. [21]found a decreased regional homogeneity in the putamen in PD patients compared to controls on resting-state fMRI, which is inconsistent with our results.

Parkinson’s disease provides an optimal model for dissociating functions of the ventral and dorsal striatum [9]. In this study, the novel finding is that the functional connectivity in the dorsal striatum, as well as in the ventral striatum, apparently differed between patients with PD and normal controls, and between PD ON and PD OFF medication patients as revealed by resting-state fMRI.

Dopamine depletion is less in the ventral striatum than dorsal striatum in patients with PD. Thus, it has been suggested that the function of the ventral striatum may be impaired by excessive dopamine supplementation. After administration of dopaminergic medication, cortex-striatum loop connectivity increased not only in the dorsal striatum seeds, but also in the ventral striatum seeds, especially in the caudate. This finding indicated the ventral striatum may have suffered a “dopamine overdose effect” and became overactive. In addition, we evaluated the anatomical plausibility of the networks through identifying striatal functional connectivity maps for the control group, and discovered that caudate is mainly involved in cognition activity. In other words, a dopamine overdose effect in the ventral striatum may impair cognitive function. This is also in line with previous resting-state fMRI study of PD which showed that a levodopa associated shift in neural oscillations may result in a change in cognitive performance in mild to moderate stage PD patients both on and off medications as compared to age-matched controls [11]. Another study suggested that the degree of treatment-mediated cognitive change is correlated with base line network activity in PD patients treated with levodopa [22].

## Conclusions

Our results documenting a PD-associated change in cortico-striatal functional connectivity parallel previous results of increased activity in PD. These results revealed that levodopa significantly changes the cognitive and motor networks of the cortico-striatal pathways. With dopamine replacement therapy, the dorsal striatum becomes sufficient and its function is enhanced, whereas the ventral striatum is overdosed and its operations are impaired. This knowledge may cause clinicians to investigate a broader range of symptoms in deciding optimal therapy.

## Supporting Information

S1 Fig Regions showing significant functional connectivity with the striatal seeds based on voxel-wise analysis in normal control subjects (one-sample t test, P <0.001, AlphaSim, K ≥6 voxels). “Hot” colors indicate increases of connectivity with the striatal seeds in normal subjects. VSi, inferior ventral striatum; VSs, superior ventral striatum; DC, dorsal caudate; VP, ventral putamen; DP, dorsal putamen.
(TIF)

S2 Fig Regions showing significant functional connectivity with the striatal seeds in PD patients off medication (one-sample t test, P <0.001, AlphaSim, K ≥6 voxels). “Hot” colors indicate increases of connectivity with the striatal seeds in PD patients off medication.
(TIF)

S3 Fig Regions showing significant functional connectivity with the striatal seeds in PD patients on medication (one-sample t test, P <0.001, AlphaSim, K ≥6 voxels). “Hot” colors indicate increases of connectivity with the striatal seeds in PD patients on medication.
(TIF)

S4 Fig Regions showing significant functional connectivity with the striatal seeds in PD ON compared with normal control subjects (two-sample t test, P <0.05, AlphaSim, K ≥85 voxles). “Hot” colors indicate increased correlation strength in PD patients on medication and “Cold” colors indicate decreased correlation strength in PD off relative to healthy aging.
(TIF)

S1 Table Distribution of the brain regions showing significant connectivity with each seed area of striatum in control group. (DOC)

S2 Table Distribution of the brain regions showing significant connectivity with each seed area from striatum in PD-OFF group. (DOC)

S3 Table Distribution of the brain regions showing significant connectivity with each seed area fro striatum in PD-ON group. (DOC)

S4 Table Significant connectivity difference between PD-OFF and control group. (DOC)

S5 Table Significant connectivity difference between PD-ON group and control group. (DOC)
