# Supplementary figures and images for: Altered Resting-State Functional Connectivity of the Striatum in Parkinson's Disease after Levodopa Administration
Source: PLoS One. 2016 Sep 9;11(9):e0161935. doi: 10.1371/journal.pone.0161935 (PMC5017636; doi:10.1371/journal.pone.0161935)

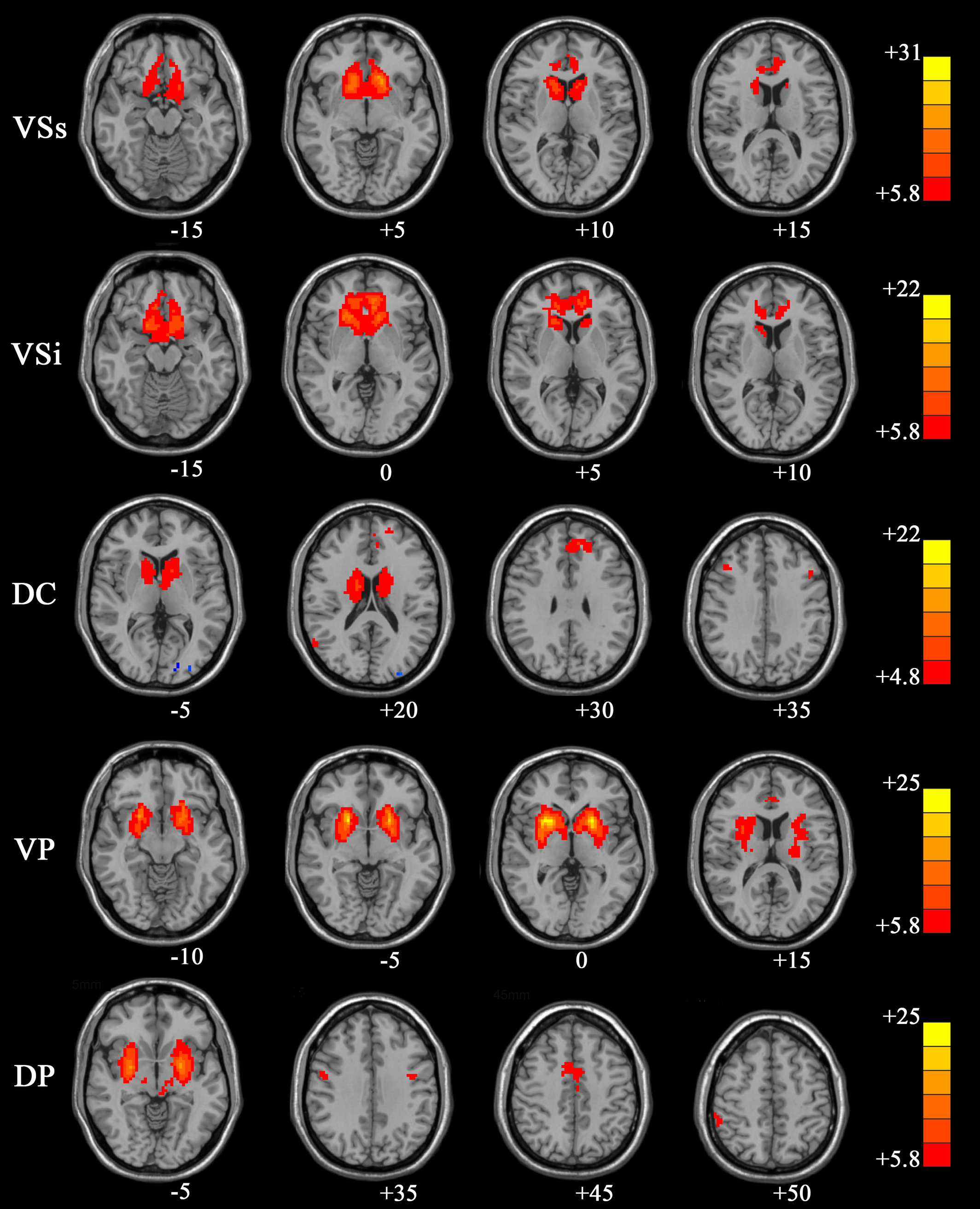

Supplement: S1 Fig — “Hot” colors indicate increases of connectivity with the striatal seeds in normal subjects. VSi, inferior ventral striatum; VSs, superior ventral striatum; DC, dorsal caudate; VP, ventral putamen; DP, dorsal putamen. (TIF) [file pone.0161935.s001.tif]

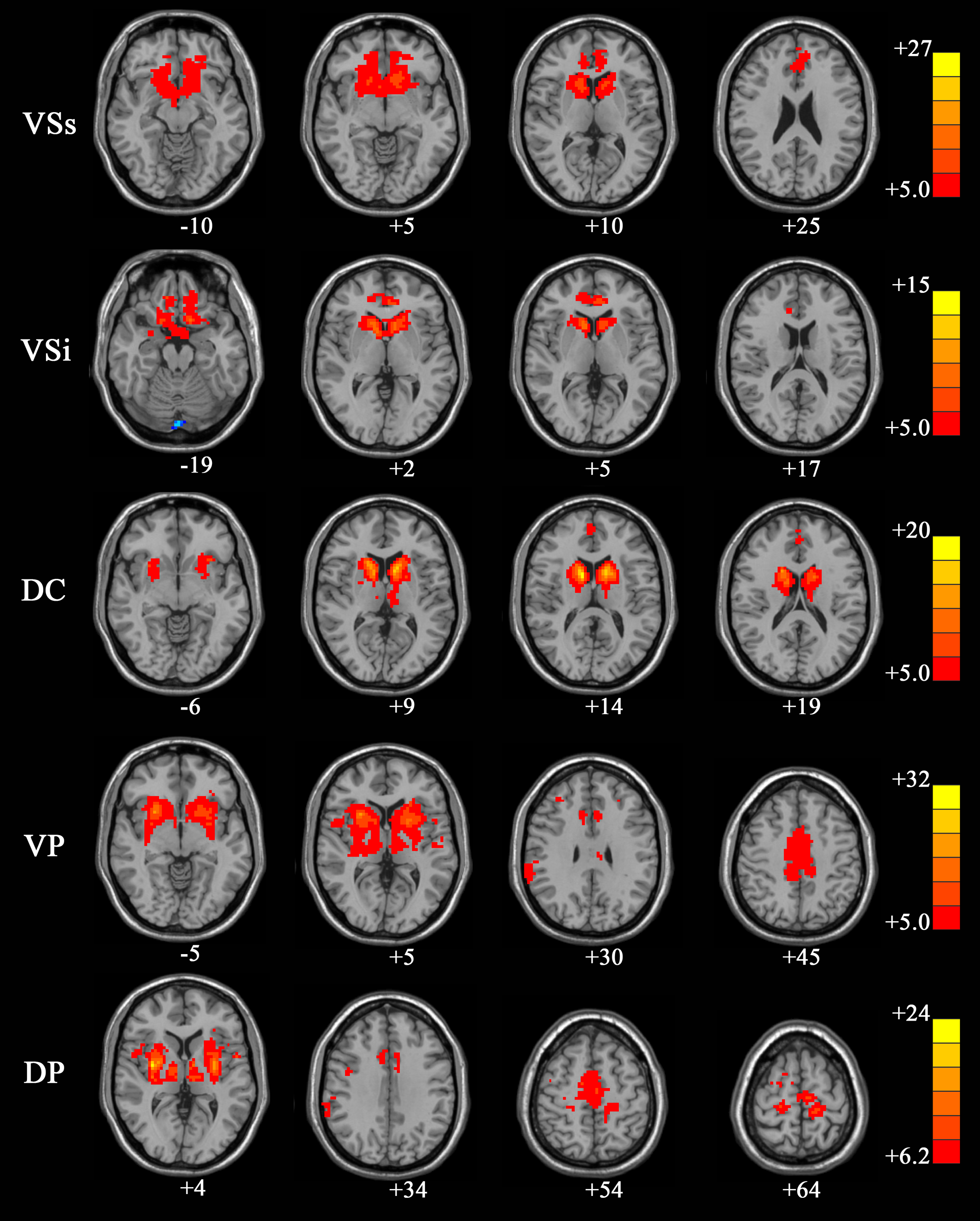

Supplement: S2 Fig — “Hot” colors indicate increases of connectivity with the striatal seeds in PD patients off medication. (TIF) [file pone.0161935.s002.tif]

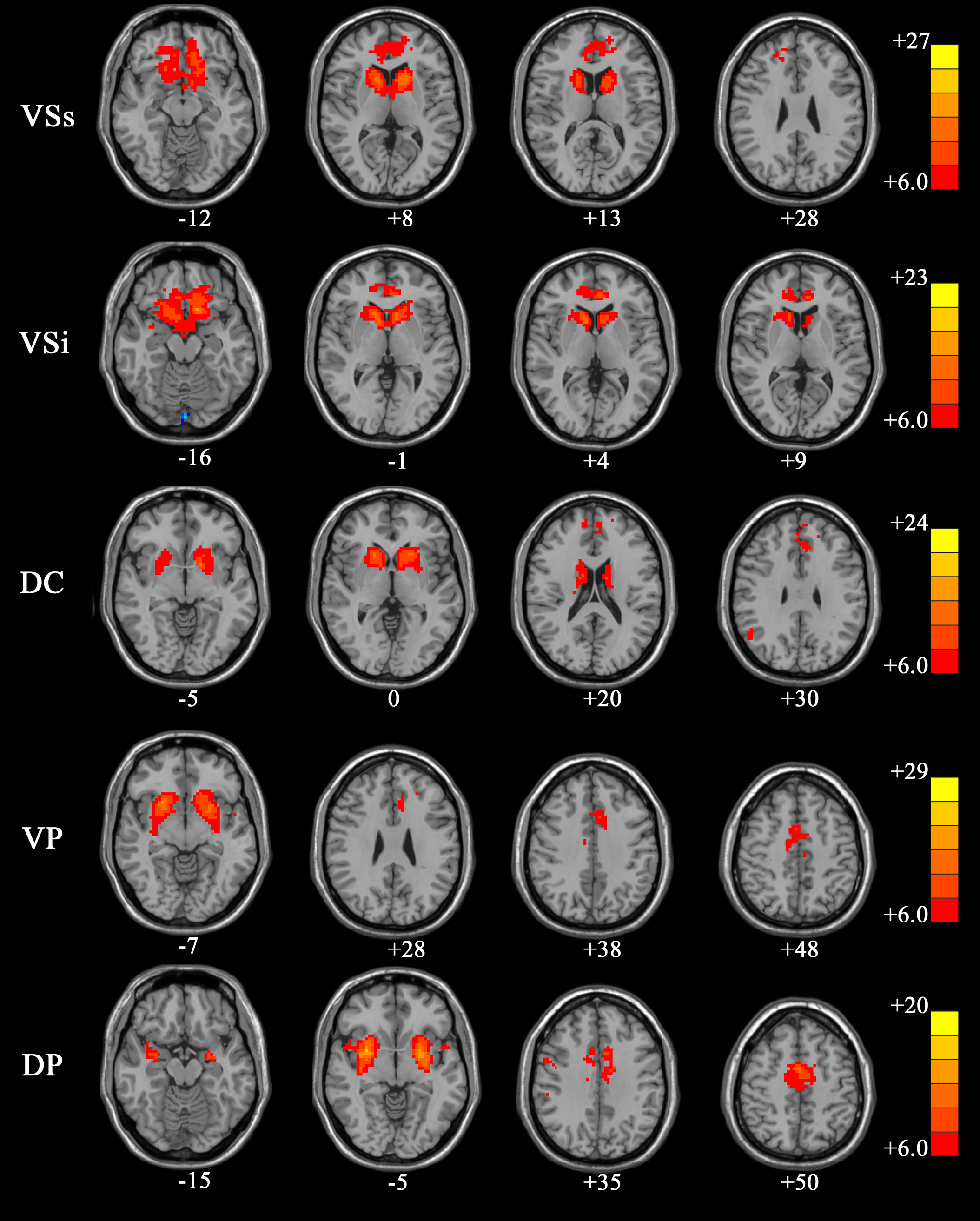

Supplement: S3 Fig — “Hot” colors indicate increases of connectivity with the striatal seeds in PD patients on medication. (TIF) [file pone.0161935.s003.tif]

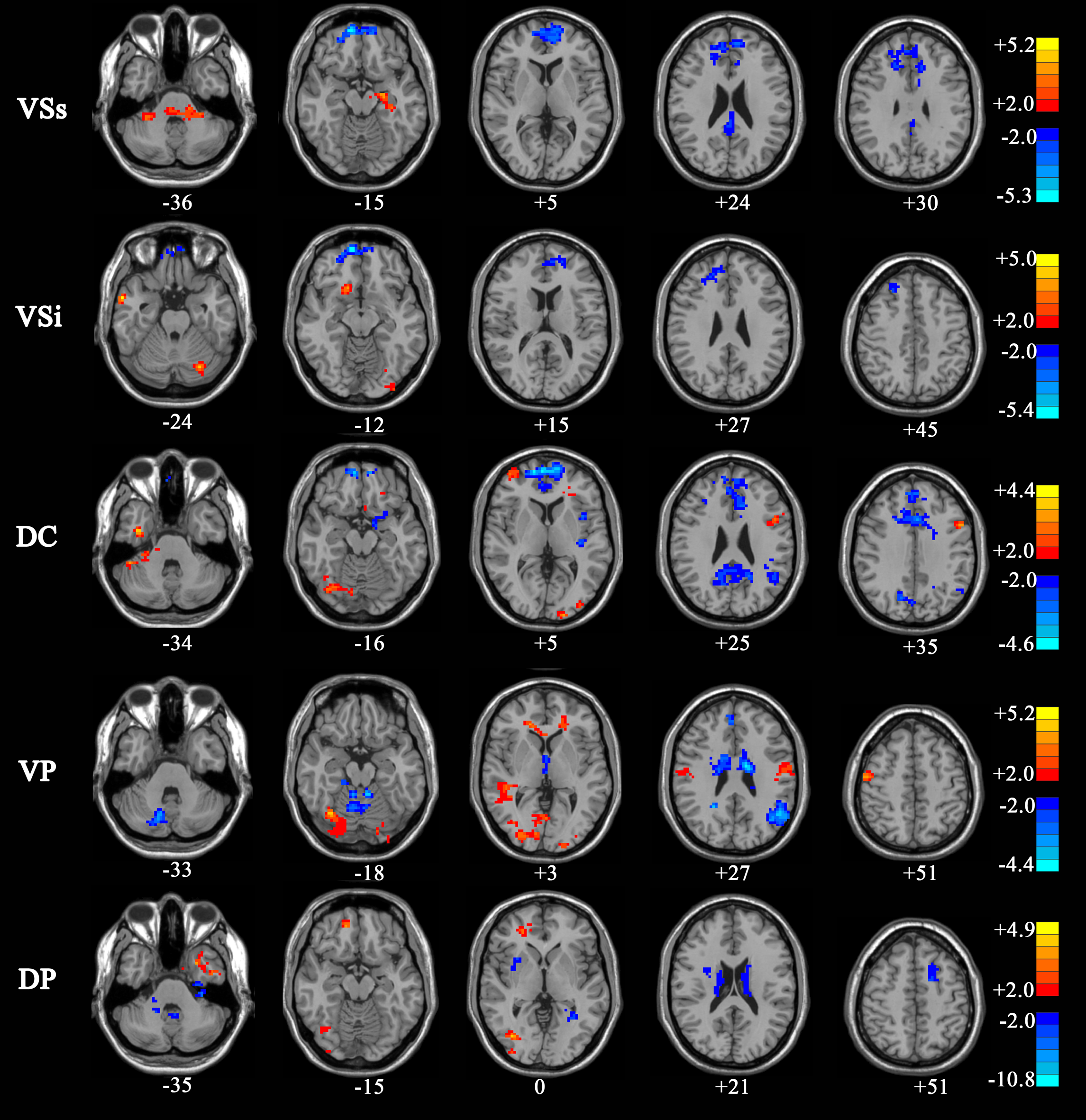

Supplement: S4 Fig — “Hot” colors indicate increased correlation strength in PD patients on medication and “Cold” colors indicate decreased correlation strength in PD off relative to healthy aging. (TIF) [file pone.0161935.s004.tif]
