# Supplementary material for: Altered Resting-State Functional Connectivity of the Striatum in Parkinson's Disease after Levodopa Administration
Source: PLoS One. 2016 Sep 9;11(9):e0161935. doi: 10.1371/journal.pone.0161935 (PMC5017636; doi:10.1371/journal.pone.0161935)
Supplement: S1 Table — (DOC) [file pone.0161935.s005.doc]

**S1 Table Functional connectivity brain area with striatum** in control group

| **Region** | **Voxel** | **MNI coordinates** | | | ***T-*value** |
| --- | --- | --- | --- | --- | --- |
|  |  | **X** | **Y** | **Z** |  |
| **Superior ventral striatum（VSs）** |  |  |  |  |  |
| Anterior Cingulate | 358 |  |  |  |  |
| Medial Frontal Gyrus | 8 | -9 | 36 | 33 | 7.8912 |
|  |  |  |  |  |  |
| **Inferior ventral striatum（VSi）** |  |  |  |  |  |
| Anterior Cingulate | 428 |  |  |  |  |
|  |  |  |  |  |  |
| **Dorsal caudate（DC）** |  |  |  |  |  |
| Caudate_R | 179 | 15 | 12 | 15 | 20.2167 |
| Caudate_L | 184 | -12 | 6 | 15 | 24.9829 |
| Medial Frontal Gyrus_L | 54 | -6 | 39 | 18 | 8.9339 |
| Superior Temporal Gyrus_R | 7 | 60 | -63 | 24 | 5.9431 |
| Superior Frontal Gyrus_L | 7 | -18 | 54 | 18 | 5.4934 |
| Superior Frontal Gyrus_R | 9 | 27 | 18 | 57 | 5.4216 |
| Middle Frontal Gyrus_R | 15 | 39 | 24 | 36 | 6.3523 |
| Middle Frontal Gyrus_L | 18 | -45 | 21 | 45 | 6.6262 |
| Cuneus_R | 6 | 18 | -78 | 39 | -5.703 |
| Frontal_Sup_Medial_R | 11 | 3 | 36 | 45 | 5.9602 |
| Frontal_Sup_Medial_L | 7 | 0 | 27 | 54 | 7.4187 |
| Supp_Motor_Area_L | 32 | -6 | 18 | 63 | 6.1515 |
|  |  |  |  |  |  |
| **Ventral Putamen（VP）** |  |  |  |  |  |
| Anterior Cingulate_R | 18 | -6 | 18 | 33 | 8.0044 |
| Precuneus_L | 10 | -3 | -66 | 45 | -8.1641 |
| Supp_Motor_Area_L | 7 | 0 | 3 | 48 | 7.4592 |
| Paracentral_Lobule_R | 11 | 6 | -42 | 72 | -8.1141 |
|  |  |  |  |  |  |
| **Dorsal putamen（DP）** |  |  |  |  |  |
| Thalamus_R | 153 |  |  |  |  |
| Thalamus_L | 135 |  |  |  |  |
| Precentral_R | 16 | 51 | -3 | 36 | 7.2924 |
| Supp_Motor_Area_R | 16 | 6 | 9 | 45 | 8.8759 |
| Parietal_Inf_R | 17 | 57 | -39 | 51 | 9.2054 |
| Paracentral_Lobule_L | 8 | -18 | -15 | 66 | 6.8959 |

Note: Distribution of the brain regions showing significant connectivity with each seed area from striatum in control group (*P* <0.001, AlphaSim, *K* ≥6 voxels). The coordinates are given as stereotaxic coordinates referring to the atlas of MNI. L, left; R, right.
