# Supplementary material for: Altered Resting-State Functional Connectivity of the Striatum in Parkinson's Disease after Levodopa Administration
Source: PLoS One. 2016 Sep 9;11(9):e0161935. doi: 10.1371/journal.pone.0161935 (PMC5017636; doi:10.1371/journal.pone.0161935)
Supplement: S2 Table — (DOC) [file pone.0161935.s006.doc]

**S2 Table Functional connectivity brain area with striatum in PD-OFF group**

| **Region** | **Voxel** | **MNI coordinates** | | | ***T*-value** |
| --- | --- | --- | --- | --- | --- |
|  |  | **X** | **Y** | **Z** |  |
| **Superior ventral striatum（VSs）** |  |  |  |  |  |
| Anterior Cingulate | 167 |  |  |  |  |
| Medial Frontal Gyrus | 25 |  |  |  |  |
|  |  |  |  |  |  |
| **Inferior ventral striatum（VSi）** |  |  |  |  |  |
| Anterior Cingulate | 91 | -3 | 39 | 6 | 9.0427 |
| Frontal_Med_Orb_R | 9 | 9 | 39 | -3 | 7.8457 |
|  |  |  |  |  |  |
| **Dorsal caudate（DC）** |  |  |  |  |  |
| Thalamus_L | 26 |  |  |  |  |
| Medial Frontal Gyrus | 6 | 3 | 54 | 15 | 7.9256 |
|  |  |  |  |  |  |
| **Ventral Putamen（VP）** |  |  |  |  |  |
| Temporal_Sup_R | 6 | 48 | -24 | 0 | 6.5021 |
| SupraMarginal_R | 20 | 66 | -42 | 30 | 8.0187 |
|  | 6 | 66 | -30 | 39 | 7.297 |
| Cingulum_Ant_L | 6 | -6 | 18 | 30 | 8.1807 |
| Cingulum_Mid_L | 15 | -9 | -30 | 42 | 8.5517 |
| Cingulum_Mid_R | 90 | 3 | -12 | 48 | 10.1282 |
|  |  |  |  |  |  |
| **Dorsal putamen（DP）** |  |  |  |  |  |
| SupraMarginal_R | 35 | 60 | -27 | 36 | 7.8671 |
| Precentral_R | 24 | 42 | 0 | 33 | 8.9887 |
| Supp_Motor_Area_L | 175 | -3 | -15 | 51 | 12.4263 |
| Cingulum Gyrus | 110 | 9 | -27 | 45 | 7.3334 |
| Paracentral_Lobule_L | 80 |  |  |  |  |

Note: Distribution of the brain regions showing significant connectivity with each seed area from striatum in PD-OFF group (*P* <0.001, AlphaSim, *K* ≥6 voxels). The coordinates are given as stereotaxic coordinates referring to the atlas of MNI. L, left; R, right.
