# Supplementary material for: Altered Resting-State Functional Connectivity of the Striatum in Parkinson's Disease after Levodopa Administration
Source: PLoS One. 2016 Sep 9;11(9):e0161935. doi: 10.1371/journal.pone.0161935 (PMC5017636; doi:10.1371/journal.pone.0161935)
Supplement: S3 Table — (DOC) [file pone.0161935.s007.doc]

**S3 Table Functional connectivity brain area with striatum in PD-ON group**

| **Region** | **Voxel** | **MNI coordinates** | | | ***T*-value** |
| --- | --- | --- | --- | --- | --- |
|  |  | **X** | **Y** | **Z** |  |
| **Superior ventral striatum（VSs）** |  |  |  |  |  |
| Anterior Cingulate | 145 |  |  |  |  |
| Superior Frontal Gyrus | 10 | 15 | 48 | 24 | 7.9674 |
|  |  |  |  |  |  |
| **Inferior ventral striatum（VSi）** |  |  |  |  |  |
| Anterior Cingulate | 336 | -18 | 42 | 12 | 9.8702 |
|  |  |  |  |  |  |
| **Dorsal caudate（DC）** |  |  |  |  |  |
| Frontal_Sup_Medial_R | 28 | 9 | 51 | 18 | 8.3049 |
| Frontal_Sup_Medial_L | 22 | -6 | 45 | 24 | 7.2228 |
|  | 29 | -3 | 33 | 33 | 8.1076 |
| Angular_R | 12 | 54 | -60 | 30 | 6.8623 |
| Frontal_Sup_L | 21 | -18 | 42 | 39 | 7.2922 |
| Frontal_Sup_R | 19 | 18 | 24 | 42 | 9.0789 |
| Frontal_Mid_L | 24 | -36 | 24 | 42 | 9.6151 |
|  |  |  |  |  |  |
| **Ventral Putamen（VP）** |  |  |  |  |  |
| Pallidum_L | 60 |  |  |  |  |
| Pallidum_L | 55 |  |  |  |  |
| Supp_Motor_Area_L | 81 | 0 | 0 | 54 | 12.473 |
| Supp_Motor_Area_R | 63 |  |  |  |  |
| Cingulum_Mid_L | 7 | -6 | -18 | 45 | 7.0056 |
|  |  |  |  |  |  |
| **Dorsal putamen（DP）** |  |  |  |  |  |
| Frontal_Mid_L | 17 | -33 | 42 | 24 | 8.4269 |
| SupraMarginal_R | 9 | 54 | -24 | 30 | 9.0487 |
| Medial Frontal Gyruy | 192 | 0 | 0 | 54 | 12.597 |

Note: The distribution of the brain regions showing significant connectivity with each seed area from striatum in PD-ON group (*P* <0.001, AlphaSim, *K* ≥6 voxels). The coordinates are given as stereotaxic coordinates referring to the atlas of MNI. L, left; R, right.
