# Supplementary material for: Altered Resting-State Functional Connectivity of the Striatum in Parkinson's Disease after Levodopa Administration
Source: PLoS One. 2016 Sep 9;11(9):e0161935. doi: 10.1371/journal.pone.0161935 (PMC5017636; doi:10.1371/journal.pone.0161935)
Supplement: S4 Table — (DOC) [file pone.0161935.s008.doc]

**S4 Table Significant connectivity difference between PD-OFF and control group**

| **Region** | **Voxel** | **MNI coordinates** | | | ***T-*value** |
| --- | --- | --- | --- | --- | --- |
|  |  | **X** | **Y** | **Z** |  |
| **PD off＞Control** | | | | | |
| **Superior ventral striatum（VSs）** |  |  |  |  |  |
| Cerebellum Posterior Lobe-L | 43* | -21 | -39 | -54 | 3.8896 |
|  | 36* | -9 | -81 | -27 | 4.5253 |
| Cerebellum Posterior Lobe_R | 62* | 27 | -45 | -39 | 4.7141 |
| Supp_Motor_Area_L | 67* | 0 | 33 | 60 | 4.0064 |
|  |  |  |  |  |  |
| **Inferior ventral striatum（VSi）** | - | - | - | - | - |
|  |  |  |  |  |  |
| **Dorsal caudate（DC）** |  |  |  |  |  |
| Cerebellum Anteria Lobe-L | 95 | 0 | -48 | -63 | 3.1471 |
| Cerebellum Posterior Lobe-R | 119 | 48 | -63 | -42 | 3.8552 |
|  |  |  |  |  |  |
| **Ventral putamen（VP）** |  |  |  |  |  |
| Lingual_R | 32* |  |  |  |  |
| Cerebelum_Crus1_R | 30* | 30 | -84 | -21 | 4.2557 |
| Superior Frontal Gyrus | 139 | 0 | 48 | 45 | 6.0067 |
|  |  |  |  |  |  |
| **Dorsal putamen（DP）** |  |  |  |  |  |
| Occipital_Sup_L | 39* | -21 | -78 | 30 | 4.3647 |
| Thalamus_L | 38* | -18 | -30 | 3 | 4.4015 |
| Parietal_Inf_L | 70* | -42 | -48 | 48 | 3.4307 |
|  |  |  |  |  |  |
| **PD off＜Control** | | | | | |
| **Superior ventral striatum（VSs）** |  |  |  |  |  |
| Temporal_Sup_R | 75* | 45 | -21 | -3 | -3.4074 |
| Temporal_Mid_L | 90 | -45 | -48 | 6 | -2.833 |
| Temporal_Mid_R | 90 | 66 | -39 | 9 | -2.9607 |
| Parietal_Sup_R | 58* | 27 | -48 | 60 | -4.8733 |
| Parietal_Sup_L | 134 | -18 | -42 | 57 | -4.0096 |
|  |  |  |  |  |  |
| **Inferior ventral striatum（VSi）** |  |  |  |  |  |
| Temporal_Inf_R | 9（88） | 42 | -72 | -3 | -3.4218 |
| Temporal_Sup_R | 111 | 48 | -21 | -3 | -3.8555 |
| Temporal_Mid_R | 215 |  |  |  |  |
| Temporal_Mid_L | 110 | -45 | -45 | 6 | -3.775 |
| Parietal_Sup_R | 63* | 27 | -48 | 60 | -4.1217 |
|  |  |  |  |  |  |
| **Dorsal caudate（DC）** |  |  |  |  |  |
| Thalamus_L | 18* | 3 | -33 | 15 | -3.4071 |
| Cuneus_R | 30* | 9 | -75 | 33 | -3.0911 |
| Cingulum_Mid | 87 | 3 | -30 | 39 | -3.4582 |
|  |  |  |  |  |  |
| **Ventral putamen（VP）** |  |  |  |  |  |
| Precentral_R | 242 | 21 | -21 | 69 | -5.3562 |
|  |  |  |  |  |  |
| **Dorsal putamen（DP）** |  |  |  |  |  |
| Fusiform_L | 49* | -27 | -21 | -30 | -4.2359 |
| Supp_Motor_Area | 26* | 3 | 3 | 48 | -3.6773 |
| Paracentral_Lobule | 43* |  |  |  |  |

Note: The distribution of the brain regions showing significant connectivity difference with each seed area between PD-OFF group and control group (AlphaSim，P <0.05， K ≥85 voxles). * indicates that the cluster don’t reach the preset threshold value. The coordinates are given as stereotaxic coordinates referring to the atlas of MNI. L, left; R, right.
