# Supplementary material for: Altered Resting-State Functional Connectivity of the Striatum in Parkinson's Disease after Levodopa Administration
Source: PLoS One. 2016 Sep 9;11(9):e0161935. doi: 10.1371/journal.pone.0161935 (PMC5017636; doi:10.1371/journal.pone.0161935)
Supplement: S5 Table — (DOC) [file pone.0161935.s009.doc]

**S5 Table Significant connectivity difference between PD-ON group and control group**

| **Region** | **Voxel** | **MNI coordinates** | | | ***T-*value** |
| --- | --- | --- | --- | --- | --- |
|  |  | **X** | **Y** | **Z** |  |
| **PD on＞Control** | | | | | |
| **Superior ventral striatum（VSs）** |  |  |  |  |  |
| Pons | 147 | -6 | -36 | -39 | 4.2469 |
| Hippocampus_L | 99 | -21 | -12 | -15 | 4.0262 |
|  |  |  |  |  |  |
| **Inferior ventral striatum（VSi）** |  |  |  |  |  |
| Temporal_Mid_R | 27* | 60 | 3 | -24 | 5.0266 |
| Cerebelum_Crus1_L | 37* | -27 | -75 | -24 | 4.0372 |
|  |  |  |  |  |  |
| **Dorsal caudate（DC）** |  |  |  |  |  |
| Fusiform_R | 37* | 39 | -6 | -33 | 3.6712 |
| Cerebellum Anterior Lobe-R | 46 | 48 | -42 | -33 | 3.5005 |
| Inferior Frontal Gyrus | 24 | -21 | 42 | -3 | 3.728 |
| Occipital_Inf_R | 15 | 39 | -75 | -15 | 3.1021 |
| Occipital_Mid_L | 69 | -36 | -93 | 9 | 3.4945 |
| Frontal_Mid_R | 58 | 48 | 54 | -9 | 4.356 |
|  |  |  |  |  |  |
| **Ventral putamen（VP）** |  |  |  |  |  |
| Fusiform_R | 63 | 33 | -69 | -15 | 4.6007 |
| Temporal_Mid_R | 58 | 51 | -15 | -9 | 3.2897 |
| Calcarine_R | 83 | 9 | -75 | 6 | 4.027 |
| Postcentral_L | 110 | -63 | -9 | 21 | 3.6654 |
| Postcentral_R | 94 | 54 | -9 | 54 | 4.416 |
|  |  |  |  |  |  |
| **Dorsal putamen（DP）** |  |  |  |  |  |
| Temporal_Inf_L | 82 | -39 | -6 | -30 | 4.9302 |
| Frontal_Sup_Orb_R | 98 | 15 | 48 | -12 | 3.6974 |
| Occipital_Inf_R | 78* | 36 | -75 | 0 | 3.997 |
|  |  |  |  |  |  |
| **PD on＜Control** | | | | | |
| **Superior ventral striatum（VSs）** |  |  |  |  |  |
| Frontal_Sup_Medial | 267 | 12 | 60 | -15 | -5.4024 |
| Posterior Cingulate | 90 | 0 | -39 | 27 | -3.0531 |
|  |  |  |  |  |  |
| **Inferior ventral striatum（VSi）** |  |  |  |  |  |
| Frontal_Sup_Orb_R | 40 | 12 | 60 | -15 | -5.3953 |
|  |  |  |  |  |  |
| **Dorsal caudate（DC）** |  |  |  |  |  |
| Medial Frontal Gyrus | 533 | 12 | 60 | 3 | -4.6945 |
| Putamen_L | 42 | -18 | 6 | -9 | -4.4347 |
| Posterior Cingulate | 81 | 18 | -45 | 15 | -3.3455 |
| Angular_L | 58 | -39 | -36 | 24 | -3.1596 |
|  |  |  |  |  |  |
| **Ventral putamen（VP）** |  |  |  |  |  |
| Cerebellum Posterior Lobe-R | 130 | 18 | -75 | -30 | -4.0678 |
| Angular_L | 142 | -57 | -69 | 15 | -4.4957 |
| Medial Frontal Gyrus | 101 | 3 | 48 | 21 | -3.6006 |
|  |  |  |  |  |  |
| **Dorsal putamen（DP）** | **－** | － | － | － | － |

Note: The distribution of the brain regions showing significant connectivity difference with each seed area between PD-ON group and control group ((P <0.05，AlphaSim，K ≥85 voxles). * indicates that the cluster don’t reach the preset threshold value. The coordinates are given as stereotaxic coordinates referring to the atlas of MNI. L, left; R, right.
